# Supplementary material for: Characterization of Glossy Spike Mutants and Identification of Candidate Genes Regulating Cuticular Wax Synthesis in Barley (Hordeum vulgare L.)
Source: Int J Mol Sci. 2022 Oct 27;23(21):13025. doi: 10.3390/ijms232113025 (PMC9658550; doi:10.3390/ijms232113025)
Supplement: Supplementary file 1 [file ijms-23-13025-s001.zip › Figure S1-S3.pdf]

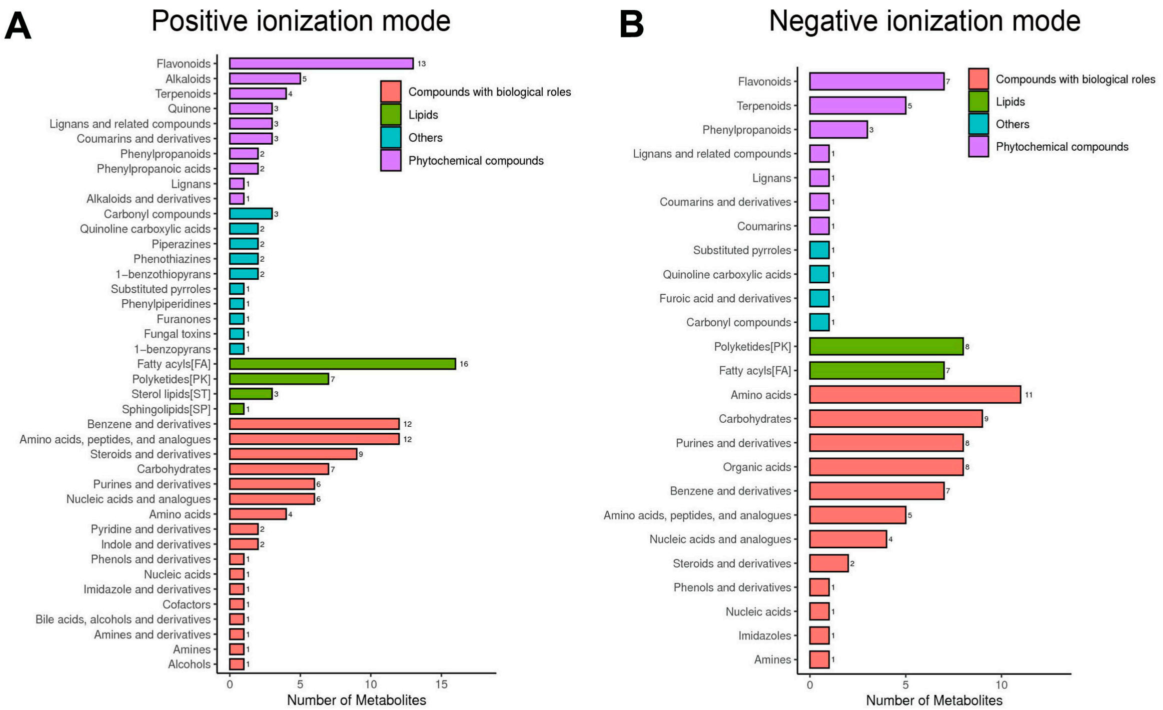

Figure S1. KEGG pathway enrichment analysis for all metabolites in the positive (A) and negative (B) ionization modes, respectively

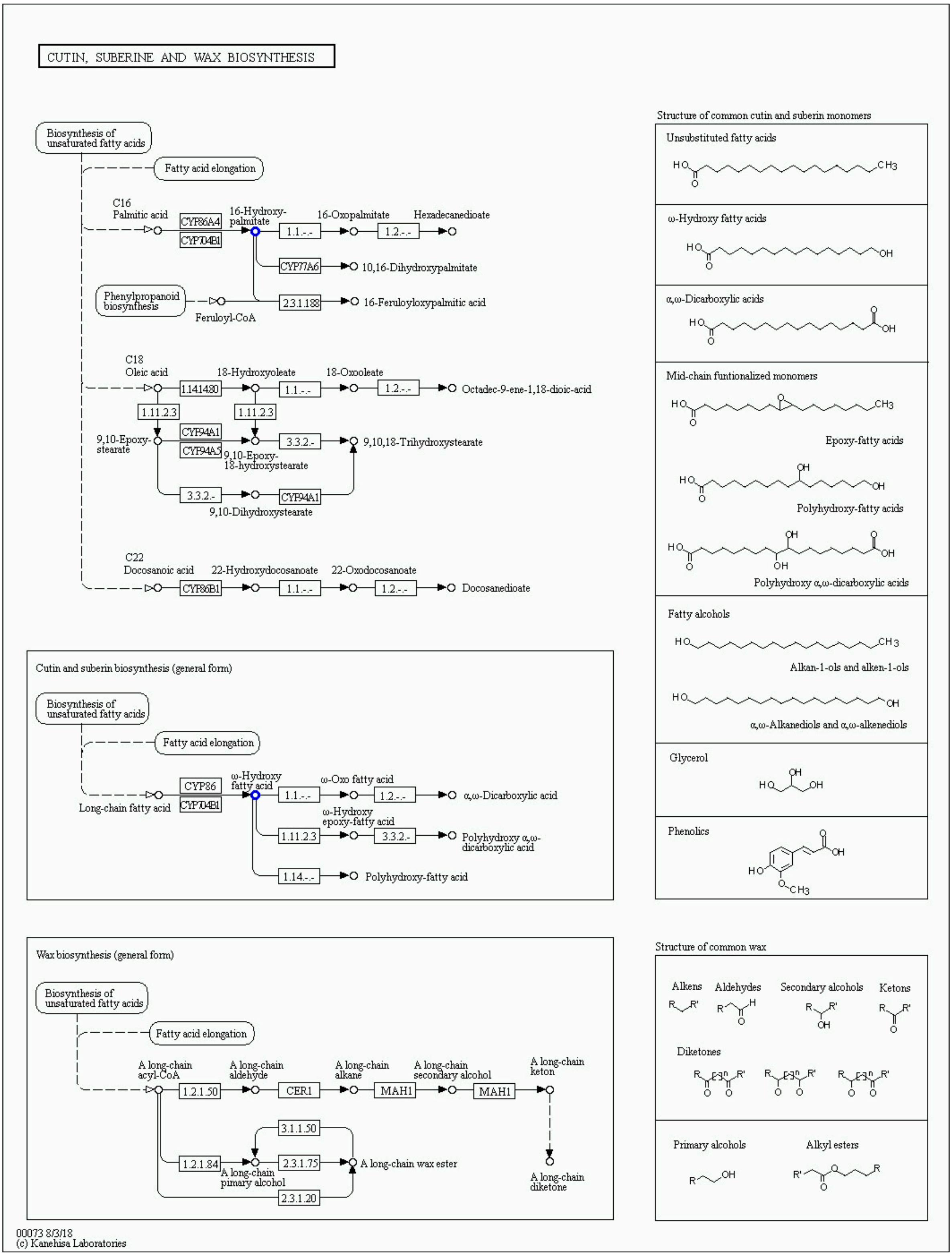

Figure S2. Metabolites involved in the KEGG pathway of cutin, suberine, and wax biosynthesis

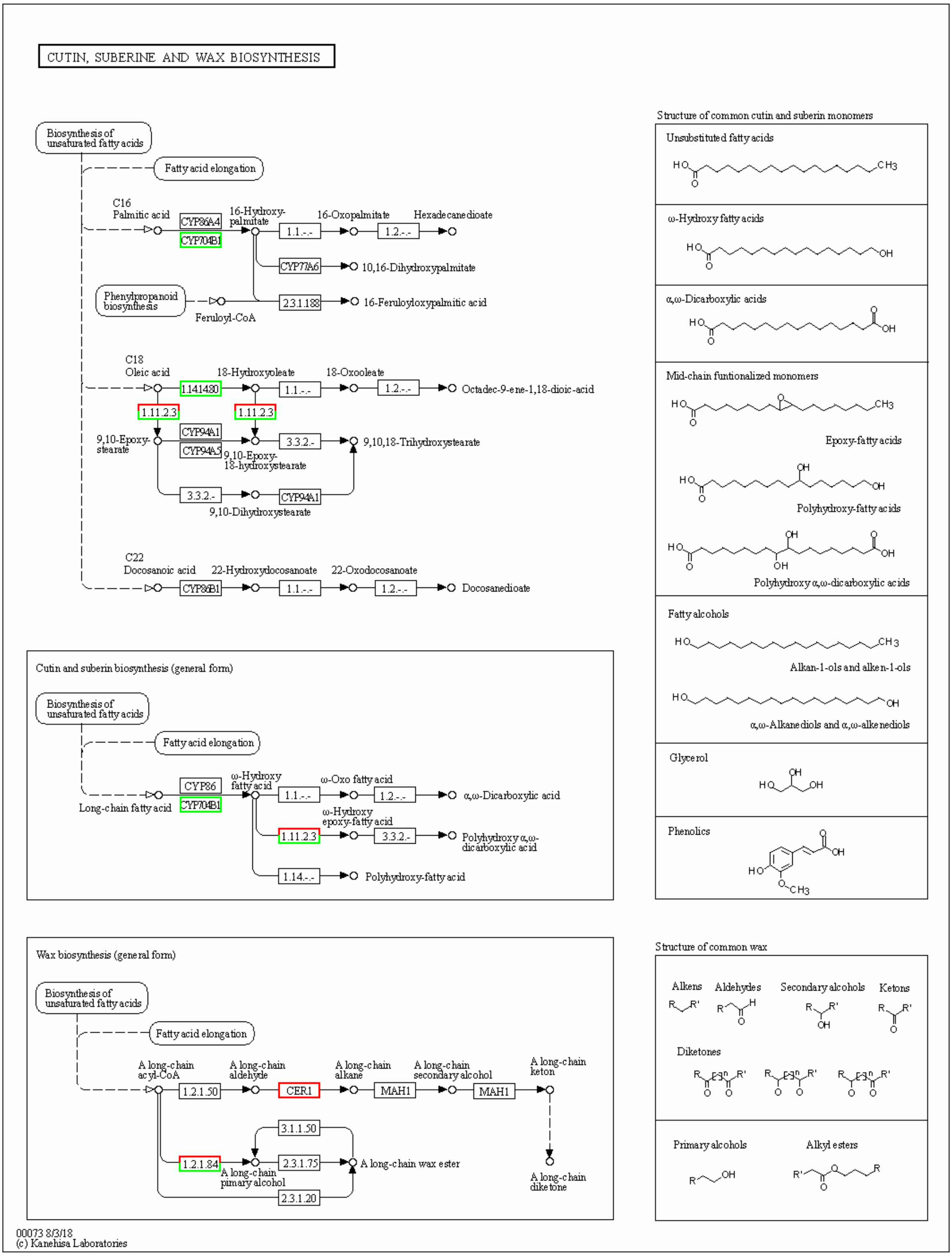

Figure S3. Genes involved in the KEGG pathway of cutin, suberine, and wax biosynthesis.
